# Comparison of anterior and posterior approaches for treatment of traumatic cervical dislocation combined with spinal cord injury: Minimum 10-year follow-up

**DOI:** 10.1038/s41598-020-67265-2

**Published:** 2020-06-25

**Authors:** Chunpeng Ren, Rujie Qin, Peng Wang, Ping Wang

**Affiliations:** 1 Department of Orthopedics, The First People’ s Hospital of Lianyungang, Xuzhou Academy of Medical Sciences, Lianyungang, China; 2 Operation-room, The First People’ s Hospital of Lianyungang, Xuzhou Academy of Medical Sciences, Lianyungang, China

**Keywords:** Diseases, Trauma

## Abstract

Anterior reduction and interbody fusion fixation has not been compared directly with posterior reduction and short-segmental pedicle screw fixation for lower cervical dislocation, and so consensus is lacking as to which is the optimal method. The purpose of this paper is to compare long-term outcomes of the anterior versus posterior approach for traumatic cervical dislocation with spinal cord injury. One hundred and fifty-nine patients could be followed for more than 10 years (follow-up rate 84.1%). Ninety-two patients underwent anterior reduction and interbody fusion and fixation, and 67 patients underwent posterior reduction and short-segmental pedicle screw fixation. Japanese Orthopaedic Association (JOA) scores, the Neck Disability Index (NDI), the American Spinal Injury Association grading (ASIA), Odom’s criteria, cervical kyphosis, operative parameters, and surgical or post-operative complications were evaluated. Patients were followed for 10 to 17 years. There was no significant difference in main JOA scores, NDI scores or ASIA scores between the two groups at follow-up. The posterior approach was associated with greater loss of alignment by two years (P = 0.012) and at final follow-up (P < 0.001). The posterior approach group had more blood loss (P < 0.001), longer operation times (P < 0.001), longer hospital stays (P < 0.001) and fewer complications than the anterior approach group. The anterior approach is better than the posterior approach for preserving cervical lordosis, which is associated with a better long-term effect.

## Introduction

Lower cervical dislocation with locked facets is common in acute cervical injury. This often leads to the abnormal alignment of the cervical spine, cervical instability and significant functional disability^1–3^. Such injury often requires early surgical treatment, with the goal of decompression and reduction. The surgical approaches for lower cervical fracture-dislocation are highly variable and include anterior, posterior, and combined anterior and posterior approaches^4–11^.

The anterior approach surgery is the most commonly used method, perhaps because it is relatively simple, is familiar to surgeons, and has achieved good results^4,5,11^. More importantly, anterior decompression is necessary for patients with disc herniation. However, in some cases, anterior reduction is difficult and also requires posterior reduction^4,12–15^. Reduction is easier to achieve with the posterior approach and can provide more stable fixation^7,8,16^, but whether it has a better outcome over a long period of time is unknown. Combined anterior and posterior approaches can not only adequately decompress, but also provide better stability^9^. However, the combined approach increases surgical trauma and complexity. Changes in position during surgery also increase the risk of nerve injury^17^. Therefore, anterior alone and posterior alone approaches are more common.

Kwon compared anterior cervical plate fixation with posterior lateral mass screw-plate and/or interspinous wire fixation for unilateral facet injuries with one-year follow-up^18^. Brodke showed no significant differences in alignment or neurologic recovery in the treatment of spinal cord injury between anterior and posterior approaches using a six month follow-up^19^. We performed either anterior reduction with interbody fusion fixation or posterior reduction with short-segmental pedicle screw fixation for lower cervical dislocations. It is important to determine the safest and most effective method to treat this population. The purpose of our study was to compare the radiological and clinical long-term outcomes of anterior versus posterior approaches for traumatic cervical dislocation with spinal cord injury.

## Methods

### Patients

This study protocol was approved by the ethics committee of the First People’s Hospital of Lianyungang and all procedures performed in the studies involving human patients were in accordance with the 1964 Helsinki declaration and its later amendments. All participants provided written informed consent. We retrospectively reviewed the records of 251 patients treated in our hospital for acute traumatic cervical dislocation combined with spinal cord injury. The following cases were included: unilateral or bilateral dislocation with or without facet joint fracture between C3-T1; dislocation amenable to either anterior single-level discectomy and plating or posterior single-level pedicle screw fixation and fusion; patient age ≥ 17 years; and follow-up of more than 10 years. Dislocations with the following characteristics were excluded: severe vertebral fracture treated by anterior cervical corpectomy and fusion, or severe osteoporosis treated by a posterior or combined anterior-posterior approach, which was defined by bone mineral density t-score ≤ −2.5 existing together with a fragility fracture. One hundred and eighty-nine patients met the inclusion criteria. Among them, 7 patients died; 11 were lost to follow-up; and 12 patients had incomplete data. Ninety-two patients included in this study underwent anterior reduction and interbody fusion and fixation from July 2002 to October 2008. Sixty-seven patients underwent posterior reduction and short-segmental pedicle screw fixation from October 2003 to March 2009. The choice of the two methods was based mainly on the preference of three senior surgeons in the Department of Spine Surgery. Also, patients with disc herniation or suspicious herniation were chosen for the anterior approach. Before surgery, continuous skull traction with a 3–4 kg weight was applied to each patient with the objective of cervical immobilization that was conducive to intraoperative traction or reduction.

Radiographic assessments were performed on anterior-posterior and lateral roentgenograms, computed tomography (CT), and magnetic resonance imaging (MRI). Vertebral kyphosis was measured by the Cobb method from the superior end plate of the cephalic adjacent intact vertebra to the lower end plate of the inferior dislocated vertebra. Symptoms and neurological status were evaluated using Japanese Orthopaedic Association (JOA) scores, the Neck Disability Index (NDI), the American Spinal Injury Association grading (ASIA), and Odom’s criteria.

These evaluations were performed preoperatively, early postoperatively, and 3, 6, 12 and 24 months postoperatively. After two years, clinical and neurological evaluations were performed every 1–2 years, and radiological evaluations were performed every 2–4 years.

The surgical procedures were conducted within three days after injury (average = 1.2 days).

### Surgical techniques

#### Anterior approach

After successful general anesthesia, a standard Smith-Robinson anterior cervical approach was performed in the supine position. Once adequate exposure had been obtained, a discectomy was performed. The pins of the Caspar retractor were inserted into the two vertebral bodies, and the two vertebrae were distracted using sleeved pins accompanied by skull traction. A periosteal detacher was inserted into the intervertebral space using the upper vertebral body as a fulcrum. As the inferior vertebra was gently levered up, the reduction was completed. To achieve better spinal canal decompression, the posterior longitudinal ligament was incised. After insertion of a polyetheretherketone (PEEK) Cage (Depuy Company, America; or Wego Company, China) filled with osteophyte particles removed during decompression or autogenous iliac crest, an anterior cervical plate was used for fixation.

After surgery, a hard neck collar was used to protect the cervical vertebrae for 12 weeks.

#### Posterior approach

The patient was placed in the prone position, and the head was fixed by a Mayfield head holder. An incision was made in the midline and the superior and inferior facets at the injured level were exposed bilaterally. After the locked facets were identified, the thin straight spinal curette was placed between the inferior facet of the cranial vertebra and the superior facet of the caudal vertebra. The handle of the curette was then gently pulled caudally so that the cranial facet was levered up and over the caudal facet. If the reduction was not completed, this maneuver was repeated, even the partial inferior facet was excised. Under the guidance of a C-arm X-ray, the pedicle screws were implanted manually. The dislocated segment was fixed with pedicle screws (Wego Company, China) and fused with allogeneic bone graft on the surface of the laminae and facet joints.

After surgery, a hard neck collar was used to protect the cervical vertebrae for 12 weeks.

### Statistical analysis

The clinical and radiographic records were compared using an independent two sample *t* test. A Chi-square test was used to compare frequency data, with P < 0.05 considered statistically significant for both tests.

## Results

One hundred and fifty-nine patients could be followed for more than 10 years (follow-up rate 84.1%). The average age of the 92 patients in the anterior approach group was 53.1 ± 14.2 years (range, 19 to 74 years); 63 were men and 29 were women. The average age of the 67 patients in the posterior approach group was 54.7 ± 15.6 years (range, 22 to 76 years); 44 were men and 23 were women. Patients in the anterior approach group were reviewed after an average follow-up of 13.4 years (range 10–17 years), and in the posterior approach group after an average follow-up of 12.7 years (range 10–16 years). There was no statistically significant difference between the groups with respect to age, sex distribution, follow-up times, dislocation level or preoperative degree of vertebral slip (Table 1).

**Table 1:** Demographic Data of the Patients.

	Anterior approach (n = 92)	Posterior approach (n = 67)	*P* value
Year	53.1 ± 14.2	54.7 ± 15.6	0.504
Male, n (%)	63 (68.5)	44 (65.6)	0.710
Follow-up	13.5 ± 2.3	12.8 ± 1.9	0.052
Unilateral dislocation, n (%)	62 (67.4)	42 (62.7)	0.538
Bilateral dislocation, n (%)	30 (32.6)	25 (37.3)	0.539
Degree of vertebral slip (mm)	3.7 ± 0.9	3.6 ± 0.9	0.610
**Segment of dislocation**			
C3–4, n (%)	13 (14.1)	7 (10.4)	0.489
C4–5, n (%)	27 (29.3)	22 (32.9)	0.638
C5–6, n (%)	31 (33.7)	24 (35.9)	0.781
C6–7, n (%)	19 (20.7)	12 (17.9)	0.667
C7-T1, n (%)	2 (2.2)	2 (3.0)	0.747

**Figure 1:**
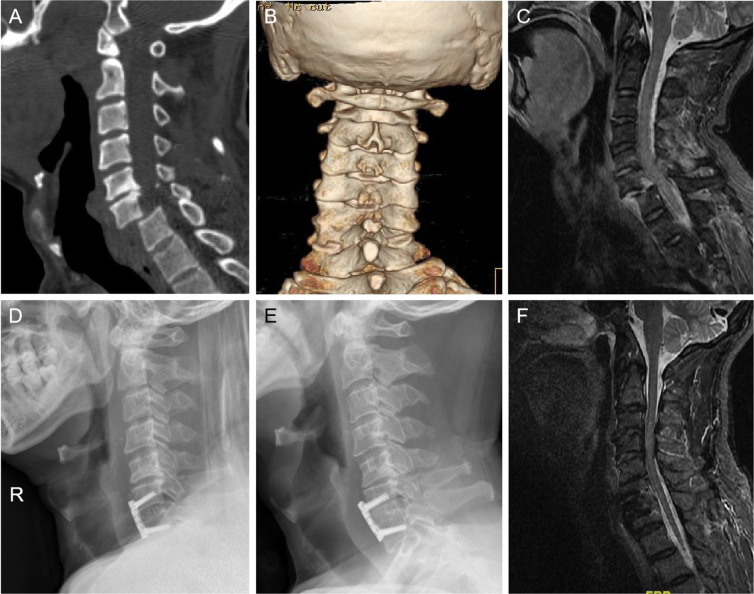
Imaging of anterior surgery. A 58-year-old man with C6/7 dislocation (**A**). 3D-CT showed the right facet was interlocked (**B**). X-Ray showed satisfactory reduction (**D**). No obvious loss of the lordosis angle in C6/7 level at 11 years after surgery (**E**). MRI showed no spinal stenosis in C6/7 level, and the spinal cord signal was normal (**F**).

**Figure 2:**
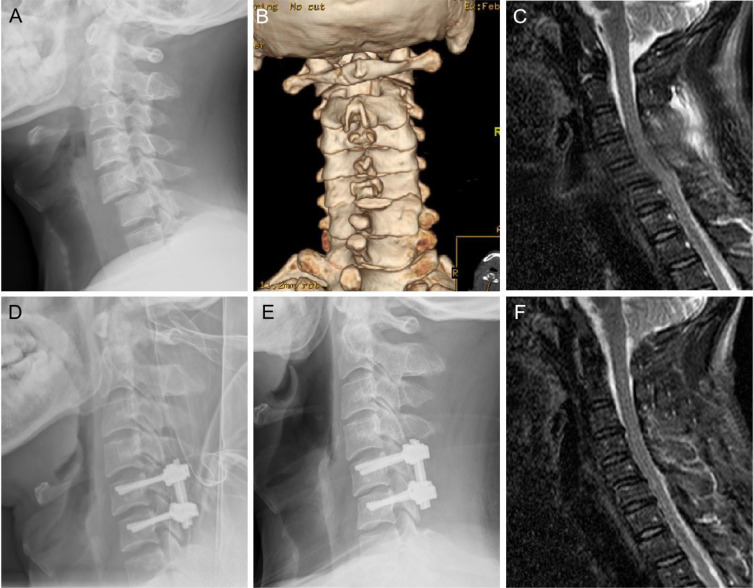
Imaging of posterior surgery. A 41-year-old man with C5/6 dislocation (**A**). 3D-CT showed the right facet was interlocked (**B**). X-Ray showed satisfactory reduction (**D**). Narrowing of intervertebral space and loss of the lordosis angle in C5/6 level at 14 years after surgery (**E**). MRI showed no spinal stenosis in C5/6 level, and the spinal cord signal was no abnormal (**F**).

Satisfactory reductions occurred in 90 patients in the anterior group and in all 67 patients in the posterior group (Figs. 1 and 2). There were two cases of failure of anterior reduction who needed additional posterior reduction. All patients achieved solid fusion within two years after surgery. There was no significant difference in main JOA score between the two groups preoperatively (P = 0.798), at 6 months (P = 0.882), or two years (P = 0.647) postoperatively, or at final follow-up (P = 0.212). The difference in recovery rate was not statistically significant between anterior and posterior approaches (65.5 ± 89.6% vs. 64.7 ± 54.5%; P = 0.951) (Table 2). The NDI in the posterior group was lower than in the anterior group at final follow-up (P = 0.015), but the recovery rate was not statistically different (P = 0.402). There were no differences preoperatively (P = 0.326), or at 6 months (P = 0.550) or two years (P = 0.148) postoperatively.

**Table 2:** Group statistics on clinical and radiological outcomes.

	Anterior approach	Posterior approach	*P* value
**JOA/recovery rate**			
Preoperative	9.5 ± 3.6	9.6 ± 3.4	0.798
1/2 y	12.4 ± 3.1	12.3 ± 2.8	0.882
2 y	13.8 ± 2.0	13.9 ± 1.6	0.647
Final follow-up	14.2 ± 1.6	14.4 ± 1.0	0.212
Recovery rate	65.5 ± 89.6	64.7 ± 54.5	0.951
**NDI/recovery rate**			
Preoperative	29.8 ± 8.2	28.6 ± 6.3	0.326
1/2 y	8.9 ± 2.0	8.7 ± 1.9	0.550
2 y	7.8 ± 1.7	7.4 ± 1.5	0.148
Final follow-up	7.3 ± 1.7	6.7 ± 1.4	0.015
Recovery rate	74.6 ± 5.8	75.4 ± 6.0	0.402
**Kyphosis angle**			
Preoperative	11.8 ± 2.4	12.3 ± 2.7	0.203
Postoperative	−4.4 ± 3.1	−3.8 ± 3.0	0.266
1/2 y	−3.9 ± 2.8	−3.2 ± 2.7	0.107
2 y	−3.6 ± 2.6	−2.6 ± 2.6	0.012
Final follow-up	−3.5 ± 2.6	−1.8 ± 2.9	0.000
**ASIA score/recovery rate**			
Preoperative	3.1 ± 1.1	3.2 ± 1.1	0.512
1/2 y	4.0 ± 1.0	4.1 ± 1.1	0.376
2 y	4.5 ± 0.8	4.5 ± 1.0	0.966
Final follow-up	4.6 ± 0.8	4.7 ± 0.7	0.631
Recovery rate	64.4 ± 62.5	59.5 ± 57.7	0.515
**Odom’s Criteria**			
Excellent outcome	53	36	0.533
Good outcome	27	20	0.945
Satisfactory outcome	11	10	0.585
Poor outcome	1	1	0.821
Complication	29	8	0.004
Odynophagia	19	1	0.000
Hoarseness	2	—	
Dysphagia	3	—	
Neck pain	3	6	0.125
Screw loosening	2	0	
Would infection	0	1	
Operation time	72.1 ± 9.2	93.0 ± 11.3	0.000
Blood loss	71.5 ± 14.6	102.4 ± 18.5	0.000
Length of stay	8.6 ± 1.5	13.4 ± 2.3	0.000

JOA, Japanese Orthopaedic Association; NDI, Neck Disability Index; ASIA, American Spinal Injury Association; Y, year.

The ASIA grade (A – D) was converted to a numeric score (1–4). The differences in ASIA score between groups was not statistically significant before surgery, at 6 months or two years postoperatively, or at final follow-up (Table 2). Six patients with ASIA grade A in the anterior group and two patients with ASIA grade A in the posterior group used a respirator after surgery. They were removed successfully from the respirator within one month.

Finally, according to Odom’s criteria, 80 patients (87.0%) in the anterior group and 56 (83.6%) in the posterior group had good to excellent clinical outcomes (P > 0.05) (Table 2).

There was no significant difference in kyphosis between the two groups preoperatively, or six months after surgery, but the posterior approach group had greater loss of alignment two years postoperatively (P = 0.012) and at the final follow-up (P < 0.001) (Table 2).

The posterior approach group had more blood loss (102.4 ± 18.5 ml vs. 71.5 ± 14.6 ml; P < 0.001) and longer surgical times (93.0 ± 11.3 minutes vs. 72.1 ± 9.2 minutes; P < 0.001) than the anterior approach group. The posterior approach group had considerably longer hospital stays (13.4 ± 2.3 days vs. 8.6 ± 1.5 days; P < 0.001) than the anterior approach group (Table 2).

Nineteen odynophagia cases occurred in the anterior group and one occurred in the posterior group during the early postoperative period. This symptom disappeared without special treatment after 1–2 weeks. Hoarseness was noted in two patients and dysphagia in three patients in the anterior group. Four patients described resolution of these symptoms at the 2-week follow-up, and one patient with hoarseness described resolution at the 1-month follow-up. Three patients in the anterior approach group reported postoperative neck axial pain that disappeared within one year. Six patients in the posterior approach group reported neck axial pain that disappeared within two years (Table 2). Seven patients in the anterior group and five in the posterior group had a postoperative pulmonary infection, which was cured within 23 days. Three patients in the anterior group complained of pain or numbness at the iliac crest bone donor site. All of them experienced relief 1–2 months after surgery.

At three months follow-up, two case of bilateral dislocation in the anterior group had screw loosening at the C6/7 level, which created instability and required posterior fixation.

There were no complications related to pedicle screw placement. One superficial wound infection that healed within 3 weeks occurred in the posterior group.

## Discussion

Although surgical methods for cervical dislocation are varied, many researchers believe that treatment decisions are likely to be affected by the neurologic status of the patient, interpretation of a disc herniation, and the classification of the injury as a unilateral or bilateral injury^19–21^. The training and experience of surgeons are also closely related to the choice of methods. There is an increased likelihood that a surgeon will use an anterior approach for decompression when they diagnose the presence of a preoperative disc herniation. But Abumi^16^ and Park^21^ reported the use of the posterior pedicle screw system to achieve reduction and removal of herniated disc fragments for cervical facet dislocations by a single posterior approach. This procedure achieves satisfactory reduction with no cases of neurologic deterioration. More research is needed to confirm this finding.

Surgeons tend to use more combined approaches when treating bilateral versus unilateral facet dislocations^5,9^. We used posterior pedicle screw short segment fixation without increasing surgical trauma and without more restricting the postoperative range of motion. At the same time, pedicle screw fixation provides three-column stability of the cervical spine, especially in cases of bilateral dislocation that may be more unstable^22,23^. For the first time, we compared posterior reduction and short-segmental pedicle screw fixation with anterior reduction and plate fixation for lower cervical dislocation.

A 13% incidence of radiographic loss of alignment was reported in 87 unilateral and bilateral facet fracture subluxations stabilized with anterior cervical discectomy, fusion, and plating^24^. Our study shows that the anterior approach is better than the posterior approach in restoring cervical alignment at the two year postoperative follow-up. Traction and prying during anterior reduction may relax the soft tissue around the dislocation. After successful reduction, the intervertebral space is larger and a higher cage must be implanted, which increases cervical lordosis to some extent. Although posterior fixation requires three-column fixation, the disruption of the disc after dislocation leads to the weakening of the disc supporting force.

O’Dowd pointed out that biomechanical factors favor the posterior approach to reconstruct the tension band in a single approach surgery, but the clinical results favor anterior approaches^25^. Kirzner reported patients with facet joint distraction of 3 mm or more to have a worse NDI and visual analogue score for pain after undergoing anterior cervical decompression and fusion for the treatment of cervical spine injury^26^. Our study shows that satisfactory reduction and fixation can be achieved through both anterior and posterior approaches, and there are no significant differences in the improvement of JOA scores or the NDI between groups during a minimum 10-year follow-up period. Kwon reported there were no statistically significant differences in pain score, the SF-36 mental and physical scores or neurological scores in comparisons between anterior cervical plate fixation and posterior lateral mass screw-plate and/or interspinous wire fixation for unilateral facet injuries. However, the mean operating room time with the anterior approach was longer than with the posterior approach^18^.

The posterior approach group had more blood loss, longer surgical times and longer hospital stays than the anterior approach group in our study. The locked-facet can be reduced under direct visualization the posterior approach, and traction can be avoided during reduction. So, it’s easier to unlock the locked joint through the posterior approach. Some studies have reported that anterior reduction has a high failure rate and requires posterior reduction, especially for unilateral facet locking^3,4,27^. In our study, 97.8% of the patients in the anterior group received a satisfactory reduction. However, it is necessary to prolong the intraoperative traction time if reduction is difficult. The technical requirements for pedicle screw implantation are high when using the posterior approach, and this approach may take longer. The discharge criteria of each hospital are different. The main reason for the long hospitalization time for patients in our hospital is that the sutures from a posterior cervical incision cannot be removed as quickly. Kwon reported the median time for discharge was 2.75 days in the anterior group and 3.5 days in the posterior group, but this difference was not statistically significant^18^.

Many patients have laryngopharyngeal discomfort after anterior reduction and fixation, which may be one of the disadvantages of anterior surgery, though most discomfort disappears within 1–2 weeks. Radcliff *et al*. reported a 61.5% incidence of dysphagia after anterior cervicotomy^28^. In our cases, no neurological symptoms were aggravated after anterior or posterior reduction, and there was no significant difference in the recovery of neurological function. However, the anterior approach had a higher incidence of specific short-term throat complications. Jack reported four patients (7%) with radiographic failure, such as progressive kyphosis, which required additional posterior fixation after anterior surgical fixation for cervical spine flexion-distraction injuries^29^. Two patients had fixation failure at the C6/7 level with the anterior approach in our study. Therefore, anterior fixation alone at the cervicothoracic junction may not be strong enough, especially for bilateral dislocations. Extending the wear period of a neck collar or other posterior fixation may be needed. In our study, the short segment pedicle screw fixation did not cause more postoperative neck pain (P = 0.125). Similarly, Brodke reported no significant difference in neck pain when comparing anterior cervical plate fixation with posterior lateral mass screw-plate fixation for cervical spinal cord injuries during a minimum 6-month postoperative follow-up period^19^.

## Limitations

First, this study was a single center retrospective analysis. Second, there was a selection bias in inclusion criteria. For most cases of cervical dislocation, although only anterior or posterior approaches can achieve satisfactory results, there are still a few patients who need combined anterior and posterior surgery. So, during the treatment of cervical facet dislocation, a comprehensive consideration should be made to choose a suitable surgical plan according to the actual patient pathology, including indications of dislocation, fracture, traumatic disc herniation, or compression of the spinal cord, among other factors.

## Conclusions

Successful reduction and satisfactory neurological recovery can be achieved by either anterior or posterior approaches for traumatic cervical dislocation combined with spinal cord injury. However, the anterior approach is better than the posterior approach for restoring cervical alignment, which is associated with a better long-term effect.

## Data Availability

For accessing additional data the corresponding author can be contacted with respect to specific request.
